# Affordable Irrigation-Drainage System for Early Postoperative Adhesion and Surgical Site Infection Prophylaxis in Posterior Lumbar Surgery: A Technical Note

**DOI:** 10.7759/cureus.84274

**Published:** 2025-05-17

**Authors:** Vladimir Prandzhev, Donika I Vezirska

**Affiliations:** 1 Department of Neurosurgery, Military Medical Academy, Sofia, BGR

**Keywords:** adhesion prophylaxis, irrigation drainage system, open spine surgery, posterior lumbar surgery, spondylodiscitis, surgical site infection

## Abstract

There has been a significant surge in the number of operations in the field of lumbar spine surgery worldwide in the past few years. Despite the recent endoscopic advances in spine surgery, open posterior surgery is often a method of choice. During posterolateral approaches, deep and extensive subperiosteal dissection of the paravertebral muscles may need to be performed, which may cause tissue necrosis, putting the deep layers at risk for infection. Other factors causing surgical site infections (SSIs) include diabetes mellitus, obesity, low serum albumin levels, renal and hepatic insufficiency. Moreover, certain pathologies, such as spondylodiscitis, may benefit from the application of antiseptic solutions for topical surgical site irrigation. In this technical note, we present an original low-budget technique using a two-way Foley catheter, which is available in every operating room, to facilitate the simultaneous irrigation and drainage of the operative site.

## Introduction

Currently, the indications for lumbar fusion surgery are becoming more common, and spinal operations are on the rise globally [1]. While minimally invasive methods such as endoscopic surgery are gaining popularity, open surgery remains the workhorse approach for lumbar spine pathology. However, the ever-growing number of interventions is leading to an increasing incidence of perioperative complications such as surgical site infections (SSIs) [2]. Lumbar fusion surgery is frequently performed for degenerative spine disease, which occurs in patients with several comorbidities, such as obesity and diabetes mellitus, that are known to be prognostic factors for deep wound infections. Longer surgical time is an additional risk for infectious iatrogenic complications.

In recent years, it has been noted that the number of non-iatrogenic, de-novo, and pyogenic spinal infections has also been rising [3]. They are known to be persistent and difficult to eradicate. The management of infectious pathology, such as spondylodiscitis with associated epidural empyema, frequently requires surgical intervention, most commonly evacuation of the collection and decompressive laminectomy posterior to the level of compression. Repeated irrigation of the operative site with antiseptic or antibiotic solutions is performed intraoperatively to reduce the topical concentration of microbial organisms.

Several studies and systematic reviews have addressed SSI prophylaxis [4-7]. The most common measures with level of evidence Grade A-C are perioperative antibiotic prophylaxis, application of vancomycin powder at the surgical site, and irrigation of the operative site with antiseptic solutions such as povidone-iodine [4]. We describe an affordable and easy-to-apply technique for continuous irrigation and simultaneous drainage of the surgical site, which may aid in preventing SSIs and early postoperative epidural adhesions, as well as in treating non-iatrogenic spinal infections with topical application of antiseptic solutions.

## Technical report

Foley catheter structure description

The two-way Foley catheter is a sterile, flexible tube with an inflatable balloon at the distal tip intended to be inserted through the urethra and retained in the urinary bladder. It has double lumens, or separate channels, running down lengthwise. The wide central channel allows for the easy passive passage of liquid, usually urine, and side channels in the wall of the catheter permit the passage of liquid towards the catheter's balloon. Simply put, the structure of the two-way Foley catheter permits two parallel passages of liquids in different directions targeted at the same site.

Steps in the system preparation

**Figure 1:**
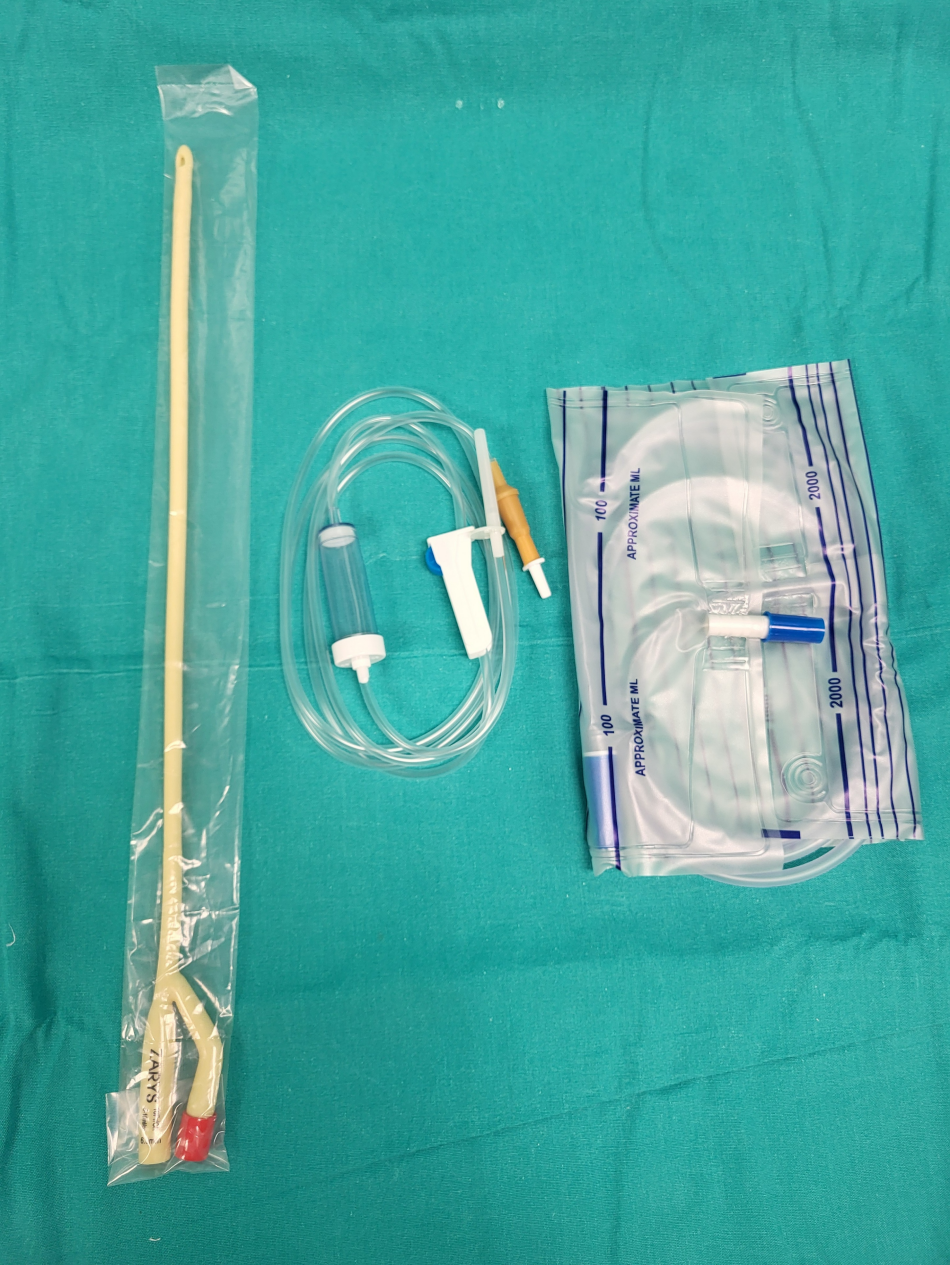
Preparation of the consumables - a two-way Foley catheter, an intravenous system set, and a drainage bag

A two-way Foley catheter, an intravenous system set, and a drainage bag are opened in a sterile manner (Figure 1).

**Figure 2:**
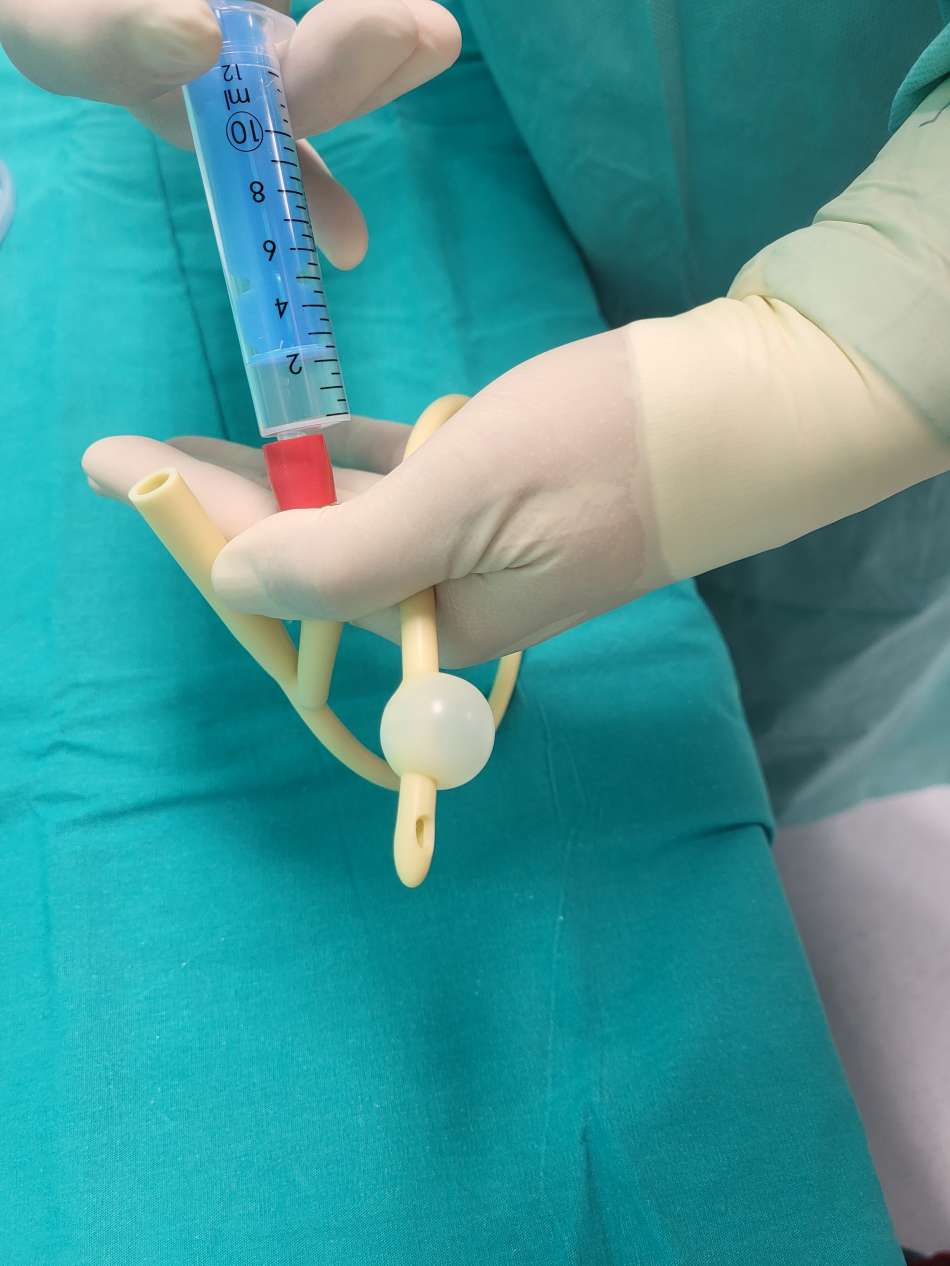
Inflating the catheter’s balloon to visualize its proximal and distal ends

The catheter’s balloon is inflated with air according to the volume indicated on the catheter to visualize the two ends of the former (Figure 2).

**Figure 3:**
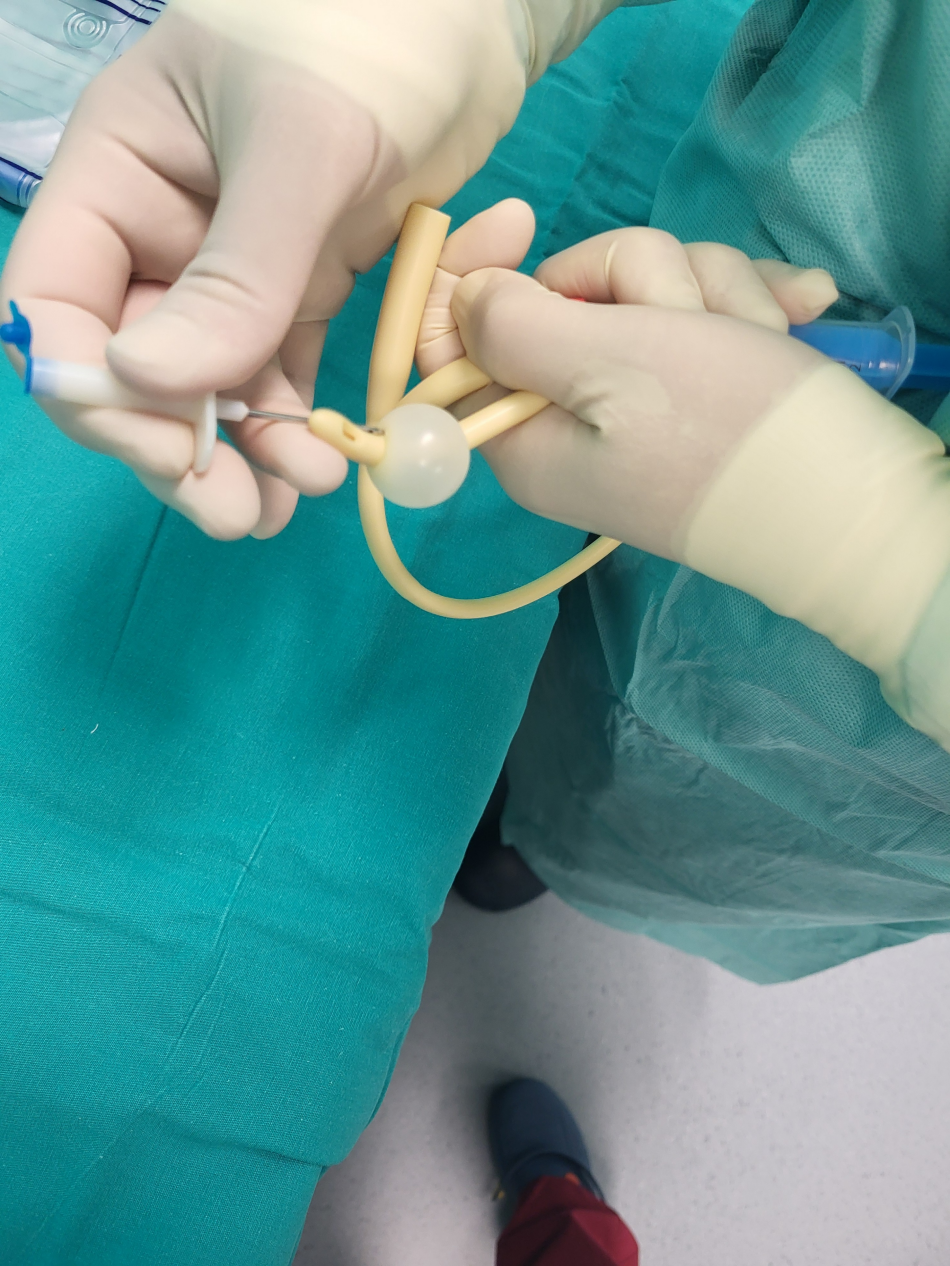
Puncturing the catheter’s balloon

The balloon is then punctured with a sterile needle (Figure 3).

**Figure 4:**
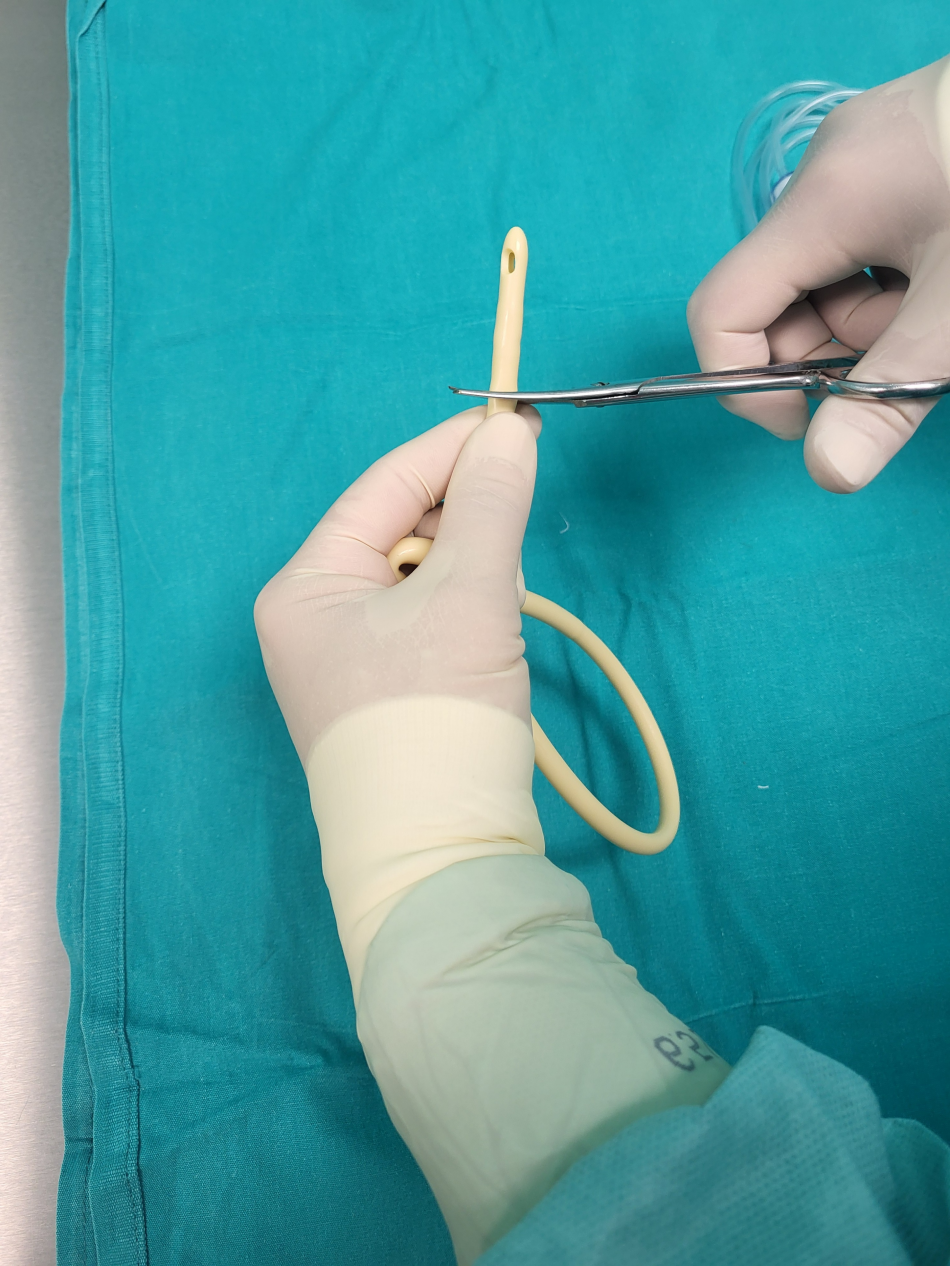
Transecting the catheter right below the balloon

The catheter is cut right before the proximal end of the balloon (Figure 4).

**Figure 5:**
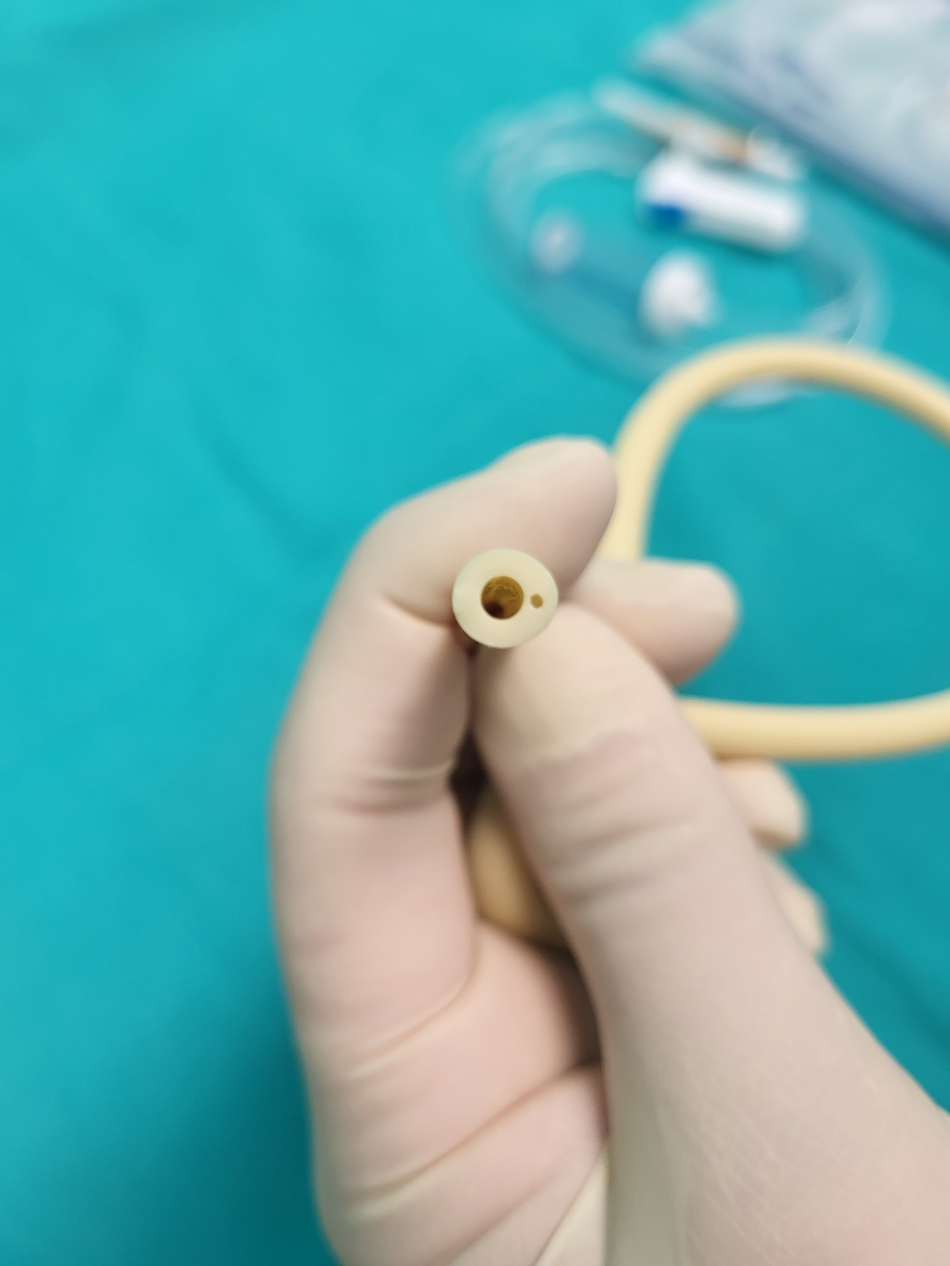
Visualization of the two channels of the Foley catheter

The two channels are visualized (Figure 5). The central lumen is used as a drainage channel, and the peripheral lumen permits the passage of continuous irrigation with an aseptic solution.

**Figure 6:**
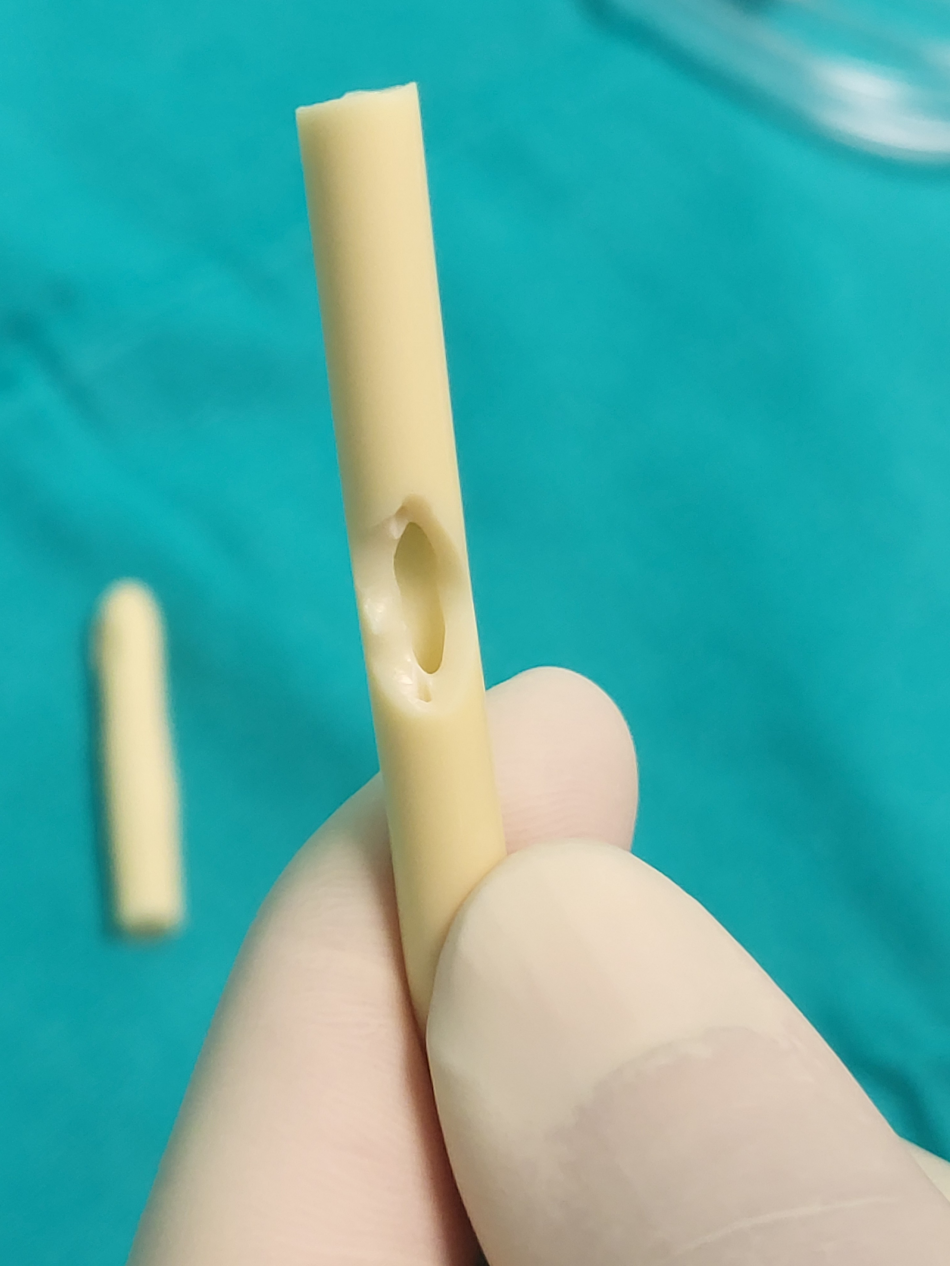
Side cuts on the catheter

Two or three side cuts are made with surgical scissors on the side of the catheter (Figure 6). The lumens should be visible.

**Figure 7:**
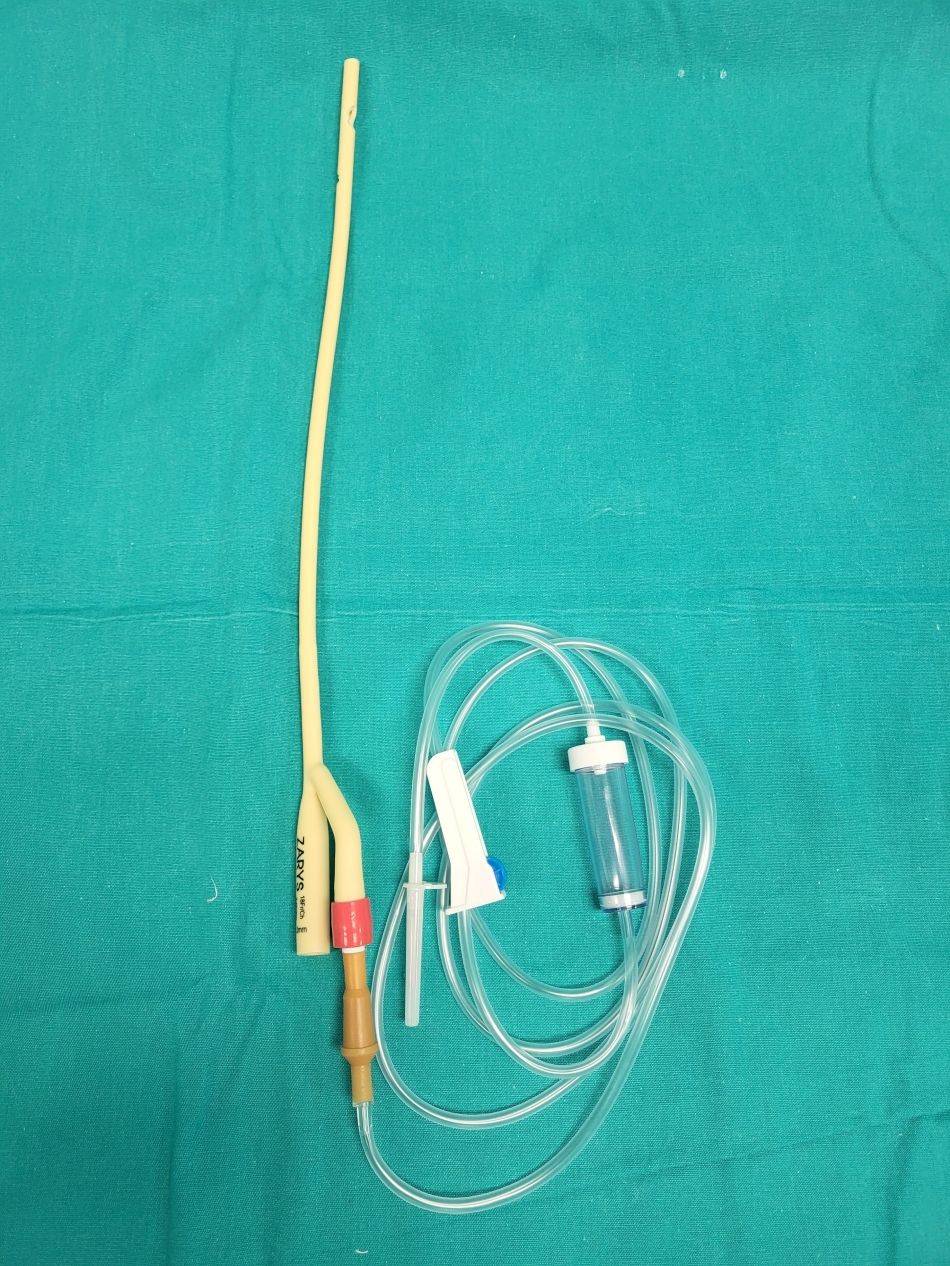
Attachment of the intravenous set to the colour-labeled lumen of the drainage bag

An intravenous set is attached to the colour-labeled lumen (Figure 7).

**Figure 8:**
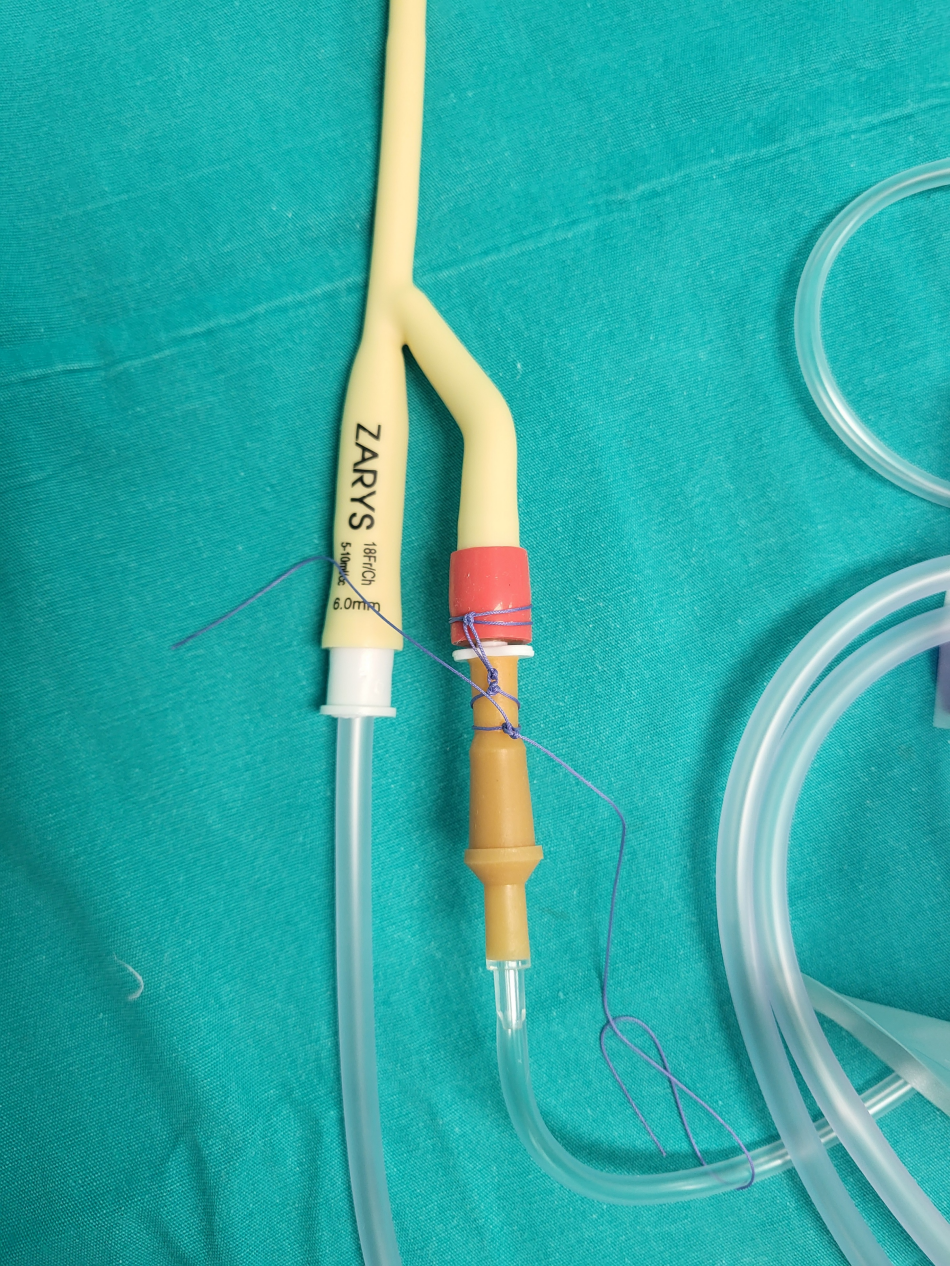
Attachment of the drainage bag and fixation of the irrigation system to the color-labeled lumen with a ligature

It is recommended that the set be fixed to the lumen with a ligature to prevent misplacement and disassembly of the system (Figure 8). A standard urine bag is attached to the proximal end of the wider lumen after the system is placed at the surgical site as a standard passive drainage system (unilaterally, around 2-3 cm from the midline surgical incision) and flushed to check if it is working properly.

In our practice, we attach a bag of 500 mL sterile saline solution (0.9% NaCl) mixed with 20 mL of povidone-iodine solution. This system is left in place for 24 hours at a moderate flow rate, as the solution bags are changed every six to eight hours. It is then extracted as a standard passive drainage tube.

Potential issues

Kinking of the drainage should be carefully straightened out to prevent blockages. Usually, this issue may occur during tunnelization of the tube when the proper angle of placement is not well planned. Additionally, we recommend placing the distal end of the catheter just above a bony edge in the superior part of the surgical site to evade epidural ulcerations while using the gravitational force to irrigate a wider area.

Postoperative issues with this system may include a lack of drainage output and difficulties with the irrigation drip. They usually occur when the patient is supine and the body weight is pressing on the tube. This may be resolved with the proper placement of the catheter on the side of the patient where the tube was inserted, while using the bridge formed by the natural lordosis curve. If this is difficult to achieve, the patient may be placed in a lateral recumbent position for the working time of the drainage system.

Another reason for the blockage may be the formation of clots. This may be resolved with a change in the irrigation flow rate and pressing of the clot site in the drainage tube over a hard surface to break it down manually.

## Discussion

SSI prophylaxis is a major issue in spine surgery, which is addressed differently by different surgeons. Yao et al. have published a systematic review on the matter, noting that the use of 0.35% povidone-iodine solution wound irrigation reduces SSI rates - this is labeled as Grade A evidence that correlates with our observations when using the described epidural irrigation-drainage system [4]. According to the study, other elements of SSI prophylaxis include perioperative antibiotic prophylaxis (Grade B evidence) and the intrawound vancomycin powder application (Grade C evidence). We also implement these steps, although this topic could be considered a research direction regarding the weight of each element in the case outcome and the SSI incidence. Other areas of possible utilization of this system may include SSI management in colorectal surgery, where it has been associated with negative economic impact, increased morbidity, extended postoperative hospital stay, readmission, sepsis, and death.

A large study of 452 treated patients who underwent a spinal instrumentation operation at a single centre was presented by Levi et al. - the treatment regimen for SSI consisted of operative debridement, a course of intravenous followed by oral antibiotics, insertion of an antibiotic-containing irrigation-suction system for a mean of five days, with no explantation of the hardware [5]. Further study that builds on the results from the latter is a large cohort of 500 patients who underwent posterior instrumented fusions [7]. The use of a closed suction irrigation system (CSIS) is noted to be effective in avoiding the need for secondary closure in postoperative deep wound infection surgery. This may imply the use of our system in iatrogenic spinal infections where SSI is already present. From our experience in such cases, after the surgical debridement, we leave the system in the surgical site for more than 24 hours and remove it upon clinical laboratory proof of a tendency for improvement in the inflammatory markers.

Hsiung et al. demonstrate the benefits of intraoperative saline irrigation in posterior lumbar surgery, suggesting that insufficient irrigation is a prognostic factor for SSI development [6]. Our technique permits postoperative continuous irrigation of the surgical site for 24 hours, which may also compensate for the insufficiency of irrigation during surgery.

According to the ERAS consensus statement, the routine use of drains in spinal surgery is not indicated [1]. However, the authors note that the evacuation of blood collections from the surgical site may prevent epidural fibrosis, a common cause of failed back surgery syndrome. While many antiadhesion barrier gels are readily available on the market, in our country, they are an additional financial burden for the patient. Seeing that failed back surgery syndrome is a pathology that is notoriously challenging to treat, this system may serve as an affordable alternative and possible adhesion prevention technique.

## Conclusions

Spinal surgery interventions are on the rise worldwide. While minimally invasive methods such as endoscopic surgery are becoming more common, open surgery remains the workhorse approach for lumbar spine pathology. Common complications of lumbar spine surgery include SSIs and postoperative epidural fibrous adhesions. This technical report presents a low-budget system that may be helpful in SSI and postoperative adhesion prophylaxis.

## References

[1] Debono B, Wainwright TW, Wang MY (2021). Consensus statement for perioperative care in lumbar spinal fusion: Enhanced Recovery After Surgery (ERAS®) Society recommendations. Spine J.

[2] Lauinger AR, Blake S, Fullenkamp A, Polites G, Grauer JN, Arnold PM (2024). Prediction models for risk assessment of surgical site infection after spinal surgery: a systematic review. N Am Spine Soc J.

[3] Zhou B, Kang YJ, Chen WH (2020). Continuous epidural irrigation and drainage combined with posterior debridement and posterior lumbar inter-body fusion for the management of single-segment lumbar pyogenic spondylodiscitis. Surg Infect (Larchmt).

[4] Yao R, Tan T, Tee JW, Street J (2018). Prophylaxis of surgical site infection in adult spine surgery: a systematic review. J Clin Neurosci.

[5] Levi AD, Dickman CA, Sonntag VK (1997). Management of postoperative infections after spinal instrumentation. J Neurosurg.

[6] Hsiung W, Yao YC, Lin HH (2023). Reducing surgical site infections after spine surgery: the optimal amount of normal saline for intra-wound irrigation. Spine J.

[7] Rohmiller MT, Akbarnia BA, Raiszadeh K, Raiszadeh K, Canale S (2010). Closed suction irrigation for the treatment of postoperative wound infections following posterior spinal fusion and instrumentation. Spine (Phila Pa 1976).

